# Uveitis including Vogt-Koyanagi-Harada syndrome following inactive covid-19 vaccination: a case series

**DOI:** 10.1186/s12348-023-00347-0

**Published:** 2023-05-19

**Authors:** Mehrdad Motamed Shariati, Mojtaba Abrishami, Shahin Jahani, Ali Bolouki, Mohamad-Reza Ansari-Astaneh, Seyedeh Maryam Hosseini

**Affiliations:** Eye Research Center, Mashhad University of Medical Sciences, Khatam Al-Anbia Eye Hospital, Qarani Blvd, Mashhad, 9195965919 Iran

**Keywords:** Coronavirus disease 2019, COVID-19 vaccination, Severe acute respiratory syndrome coronavirus 2, Sinopharm vaccine, Uveitis, Vogt-Koyanagi-Harada

## Abstract

**Background:**

Currently, large populations have been vaccinated against COVID-19. The whole inactivated Sinopharm COVID-19 vaccine has been the main available COVID-19 vaccine in Iran. Ocular inflammatory reactions have been reported following vaccination. The present case reports aim to introduce four cases of uveitis after the Sinopharm vaccine administration.

**Case presentation:**

Our first reported case is a 38-year-old woman with a positive medical history of inactive ulcerative colitis. Active uveitis had developed following the second dose of the COVID-19 vaccination. The remaining three cases were healthy individuals who developed the first episode of uveitis, after the COVID-19 vaccine administration. Vogt-Koyanagi-Harada syndrome was the final diagnosis in one of the aforementioned cases. All four patients demonstrated favorable responses to corticosteroid treatment.

**Conclusion:**

These observations are in line with incoming reports from all around the world and raise concerns about the possibility of post-vaccination uveitis development, especially in cases with a previous history of auto-immune systemic diseases or inactive uveitis.

## Introduction

COVID-19 is a contagious respiratory disease caused by severe acute respiratory syndrome coronavirus 2 (SARS-COV-2). Vaccination is one of the most effective ways to control its spread. The whole inactivated COVID-19 Sinopharm vaccine has been the main available and widely used COVID-19 vaccine in Iran.

There have been reports of inflammatory eye diseases following Sinopharm vaccination. El Sheikh et al. reported an 18-year-old female case of juvenile idiopathic arthritis with bilateral anterior uveitis after receiving a second dose of the Sinopharm vaccine [1]. Lijie Pan et al. reported a case of bilateral posterior uveitis five days after inoculation with inactivated COVID-19 vaccine with a good response to local and systemic steroids [2]. Goyal et al. reported a 34-year-old male with large macular serous detachment and choroidal thickening one week after the second dose of the COVID-19 vaccine. The patient was treated with oral corticosteroid and his condition improved rapidly [3]. It is not known whether this process is due to the vaccine triggering this immune response per se or a mere coincidence. We hereby present four cases in which uveitis developed after receiving inactivated COVID-19 vaccine.

### Case1

**Fig. 1:**
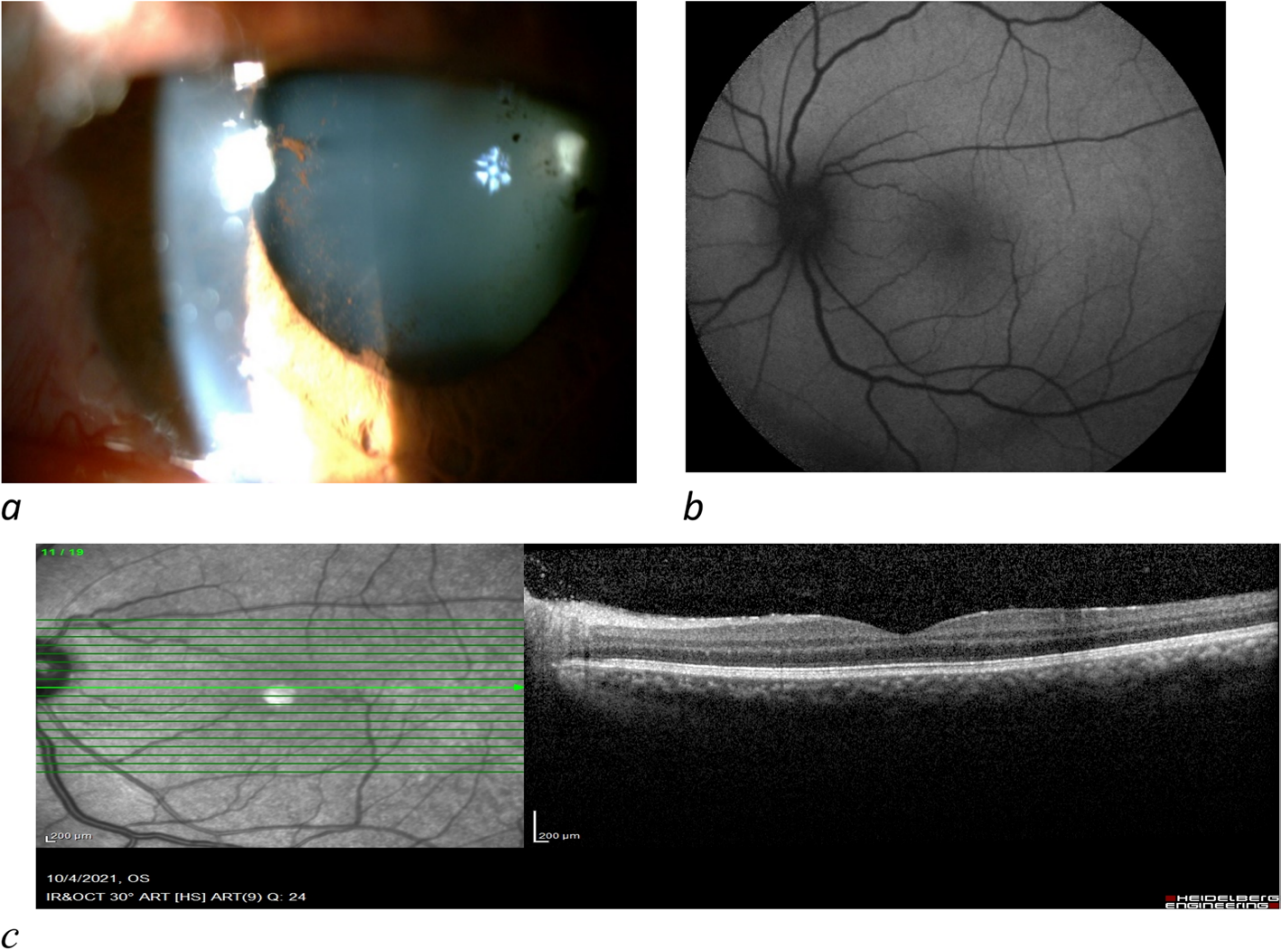
Slit photo of the left eye shows diffuse KP’s, posterior synechia, pigment on the crystalline lens, and anterior chamber reaction (**a**). FAF (**b**) and Macular OCT (**c**) of the involved eye show no abnormality

A 38-year-old woman with a history of ulcerative colitis for 20 years was referred to the emergency department of Khatam-Al-Anbia Eye Hospital, affiliated with Mashhad University of Medical Sciences, Mashhad, Iran. According to the patient’s medical history, she experienced inactive systemic disease for the last four years without any ocular manifestations albeit not taking any medications. She had complaints of blurred vision, ocular pain, and photophobia in her left eye six days after inoculation with the second dose of the Sinopharm vaccine. No other ocular or systemic symptoms were observed. The best-corrected visual acuity (BCVA) was 20/32 for her left eye. The anterior segment examination of the left eye showed posterior synechia, ciliary injection, fine keratic precipitates (KPs), 4 + anterior chamber (AC) cells and flare, and 1 + vitreous cell, according to SUN Working Group classification [4]. Intraocular pressure was within the normal limit in both eyes. Fundus examination, macular optical coherence tomography (OCT), and fundus autofluorescence (FAF) revealed no further abnormalities (Fig. 1). Rheumatologic and gastroenterology consults were requested, and treatment was started with oral prednisolone (25 mg/day), topical corticosteroid, and cycloplegic medication. The rheumatologic consult was unremarkable; however, the colonoscopy showed active ulcerative lesions. Azathioprine, mesalamine, and folic acid were initiated. After 10 days of treatment, BCVA increased to 20/20, AC reaction reduced to + 0.5, and posterior synechia was broken. Oral and topical steroids were tapered off over four weeks and no evidence of relapse was observed during a 3-month follow-up.

### Case 2

**Fig. 2:**
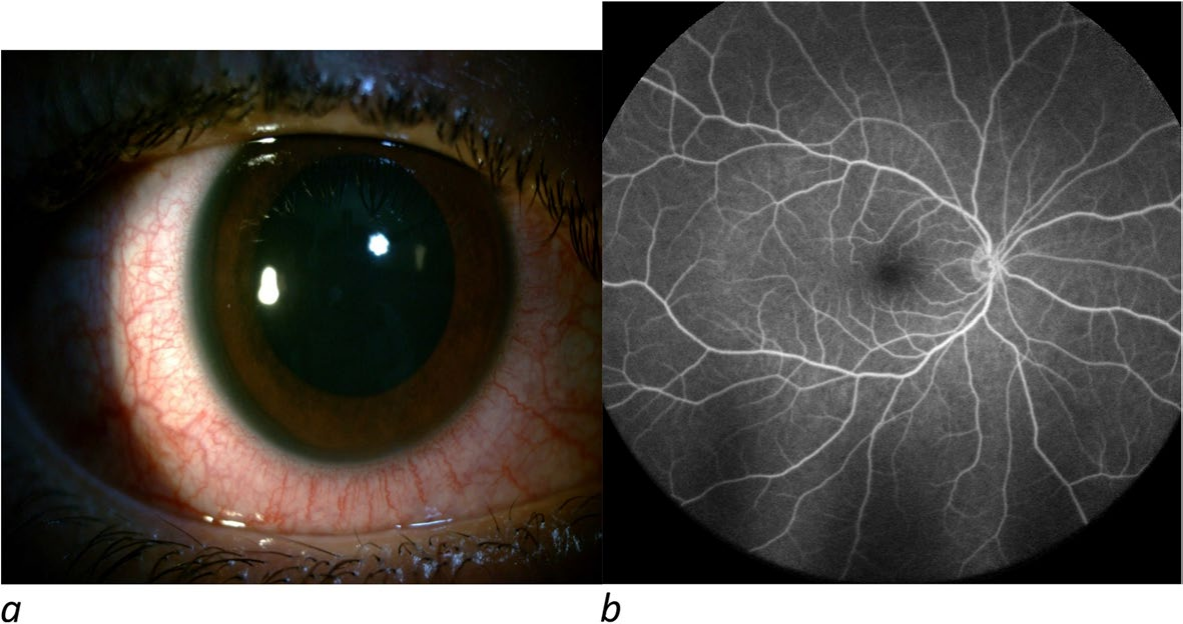
The slit photo of the Right eye shows ciliary injection (**a**). Fluorescein angiography shows no further pathology (**b**)

A 15-year-old female who developed bilateral signs and symptoms including red-eye, photophobia, and blurred vision three days after inoculation with the first dose of the Sinopharm vaccine was referred to our medical center. We examined the patient two days after the disease onset. The patient’s BCVA was 20/25 for both eyes, and slit-lamp examination revealed bilateral ciliary injection, symmetrical 4 + AC cell, and 2 + flare in both eyes. The past medical, ocular, and drug histories were unremarkable, and systemic workup results (e.g., hematologic and serologic tests) were inconclusive. Macular OCT and fluorescein angiography (FA) showed no other pathological findings (Fig. 2). We prescribed frequent topical corticosteroids and cycloplegics. After four days of treatment, the ciliary injection and AC reaction of the patient were significantly reduced and her BCVA increased to 20/20 in both eyes. Treatment was slowly tapered off within two weeks, and the inflammation decreased in the last 3-month follow-up.

### Case 3

**Fig. 3:**
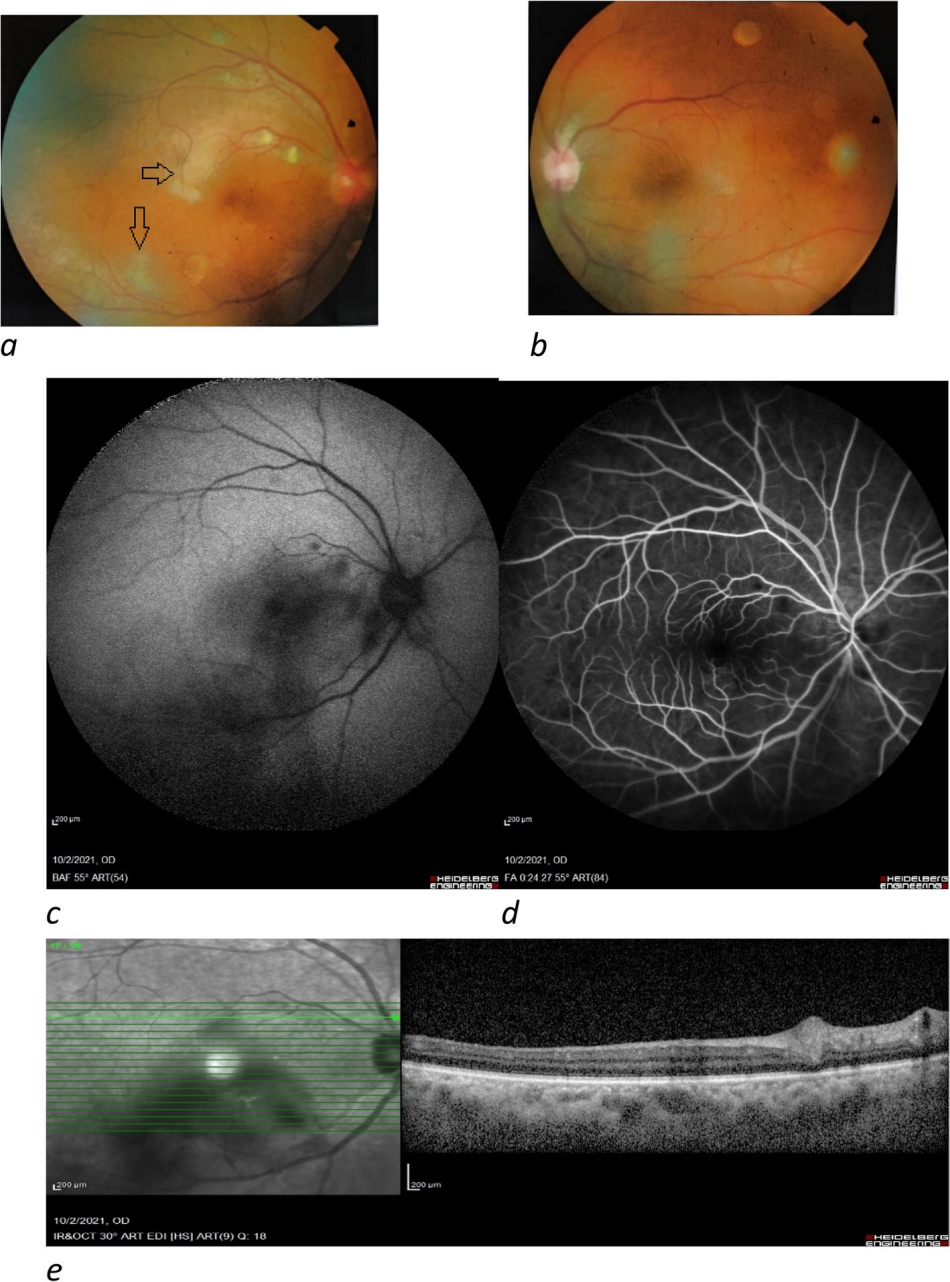
The Fundus photo of the right eye shows disc hyperemia and two small patches of retinitis; the Fundus photo of the left eye is normal (**a**, **b**). Fundus autofluorescence (FAF) shows hypo autofluorescence in the same location of retinitis patches (**c**). Fluorescein angiography showed no vascular leakage (**d**). Optical Coherence Tomography (OCT) of the right eye reveals increased inner retinal thickness and reflectivity at the site of the retinitis patches (**e**)

A 65-year-old male was referred to our clinic with complaints of blurred vision and photophobia 12 days after inoculation with the second dose of the Sinopharm vaccine. The patient reported malaise and anorexia after the injection of the second vaccine dose. Eye redness, pain, photophobia, and blurred vision occurred six days later. Slit-lamp examination showed 3 + AC cells and 1 + vitreous cells in the right eye and 2 + AC cells and no vitreous cells in the left eye. Fundus examination revealed disc hyperemia and two small patches of retinitis measuring less than two-disc diameters in the posterior pole of the right eye (Fig. 3). Fundus examination of the left eye was normal. Vitreous sampling was performed with a 25-gauge needle through pars-plana and evaluated using polymerase chain reaction (PCR) to detect the Herpes Simplex virus, Varicella Zoster virus, Cytomegalovirus, and Toxoplasma. A thorough systemic workup and rheumatology consultation was requested subsequently. Following negative results of ocular PCR for pathogens, nasopharyngeal PCR for COVID-19, and systemic work-ups oral prednisolone (25 mg/day) was started in addition to topical corticosteroid and cycloplegics. In the follow-up visit two weeks later, AC reaction reduced to 1 + cell in both eyes, and the retinitis patches were resolved. Oral and topical steroids were slowly tapered off within four weeks. Considering the results of the patient’s assessment, and the good response to steroids, immune-mediated panuveitis triggered by the COVID vaccine is suggested as the diagnosis.

### Case 4

**Fig. 4:**
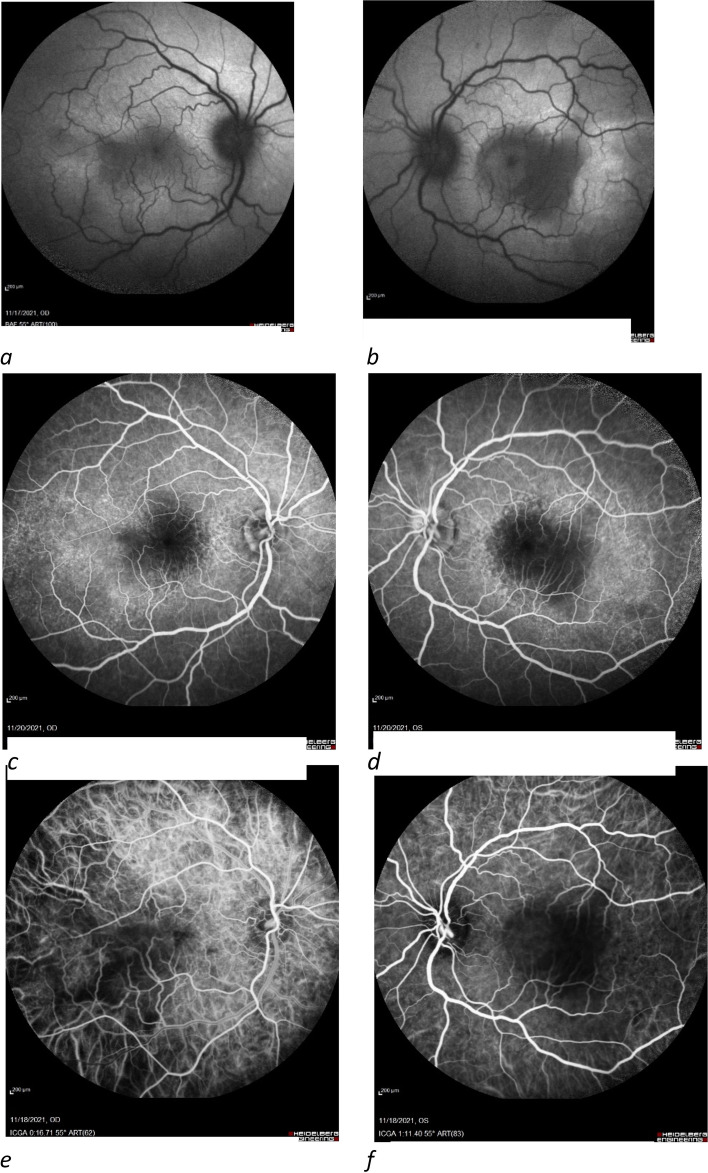
FAF shows hyper autofluorescence in the macula with hypo autofluorescence in the areas of the serous detachment (**a**, **b**); FAG reveals multiple areas of pinpoint leakage in the posterior pole (**c**, **d**). ICGA demonstrates multiple dark spots in the mid-phase angiogram (**e**, **f**)

**Fig. 5:**
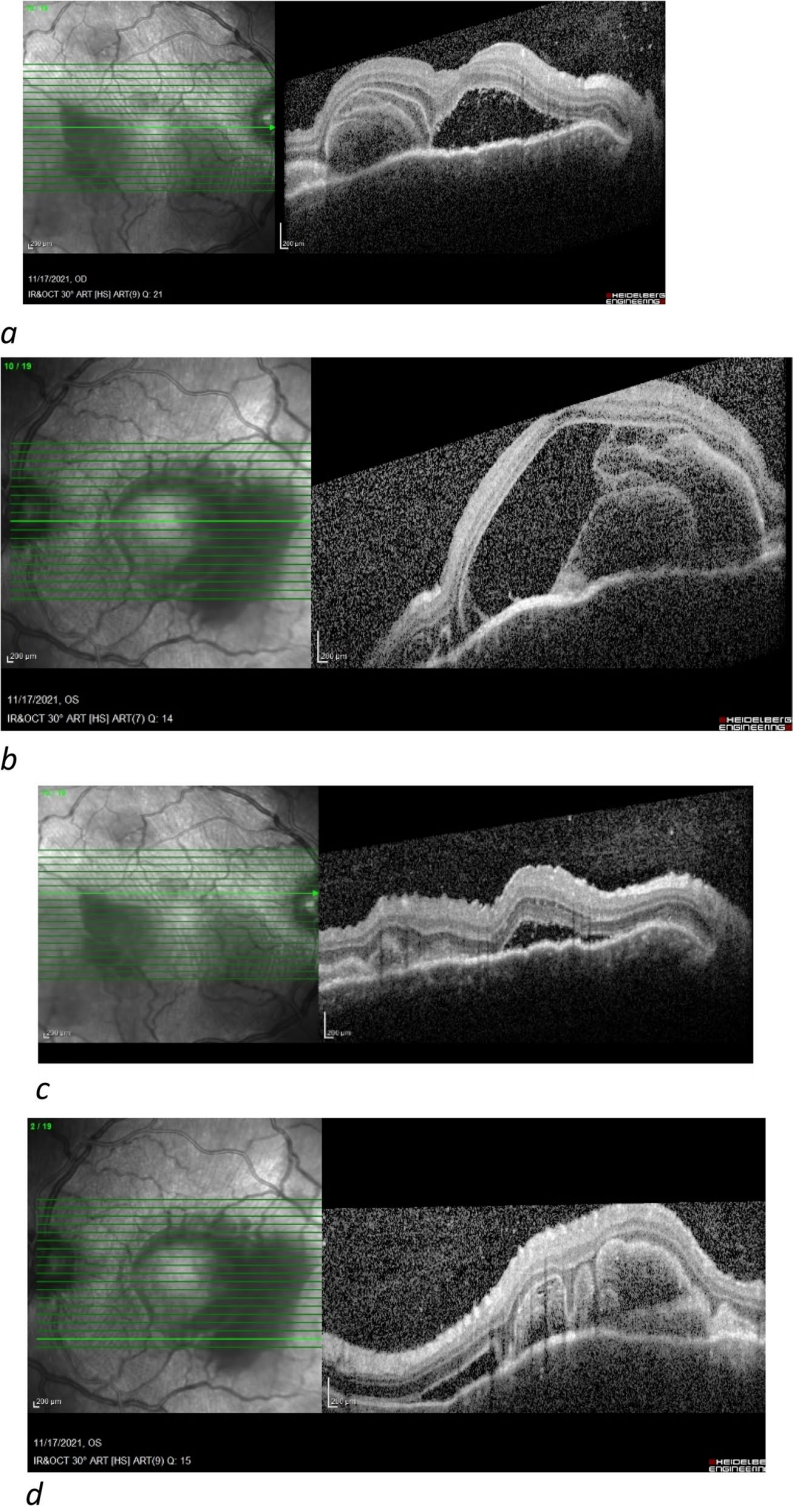
Macular OCT before treatment revealed severe serous retinal detachment in OU with multiple septae, heterogenous hyperreflective subretinal materials, and undulations of the thickened choroid. Also, severe disruption of outer retinal layers was noted (**a**, **d**)

A 23-year-old female patient with no previous medical or medication history was referred to our clinic for evaluation of progressive vision loss. She had received a second dose of the Sinopharm vaccine two weeks prior and reported episodes of malaise and fever for one week. One week after inoculation, the patient experienced severe headaches and the vision in both eyes started to decline. Topical steroid drops had been administered to her for bilateral anterior uveitis with no effect, and she was then referred to our clinic for further evaluation. BCVA was 20 /100 for the right eye and 20/630 for the left eye. Slit-lamp examinations revealed fine KPs, 3 + AC cells, 1 + flare, and 1–2 + vitreous cells in both eyes. Fundus examination showed bilateral disc hyperemia and multifocal posterior pole serous retinal detachment in both eyes. FAF, FA, and indocyanine angiography images are presented in Fig. 4. Macular OCT revealed severe serous retinal detachment in both eyes with multiple septae, heterogenous hyperreflective subretinal material, and undulations of the thickened choroid in favor of Vogt-Koyanagi-Harada (VKH) syndrome. Moreover, severe disruption of the outer retinal layers was noted (Fig. 5). The patient was admitted with the diagnosis of probable VKH syndrome, and intravenous methylprednisolone (1gr per day), topical steroid, and cycloplegic were administrated accordingly. After three days of treatment, BCVA increased to 20/50 OD and 20/500 OS, and a significant reduction of subretinal fluid was observed in the OCT images. The intravenous steroid was discontinued, and the patient was discharged with oral prednisolone (1 mg/kg/day).

**Fig. 6:**
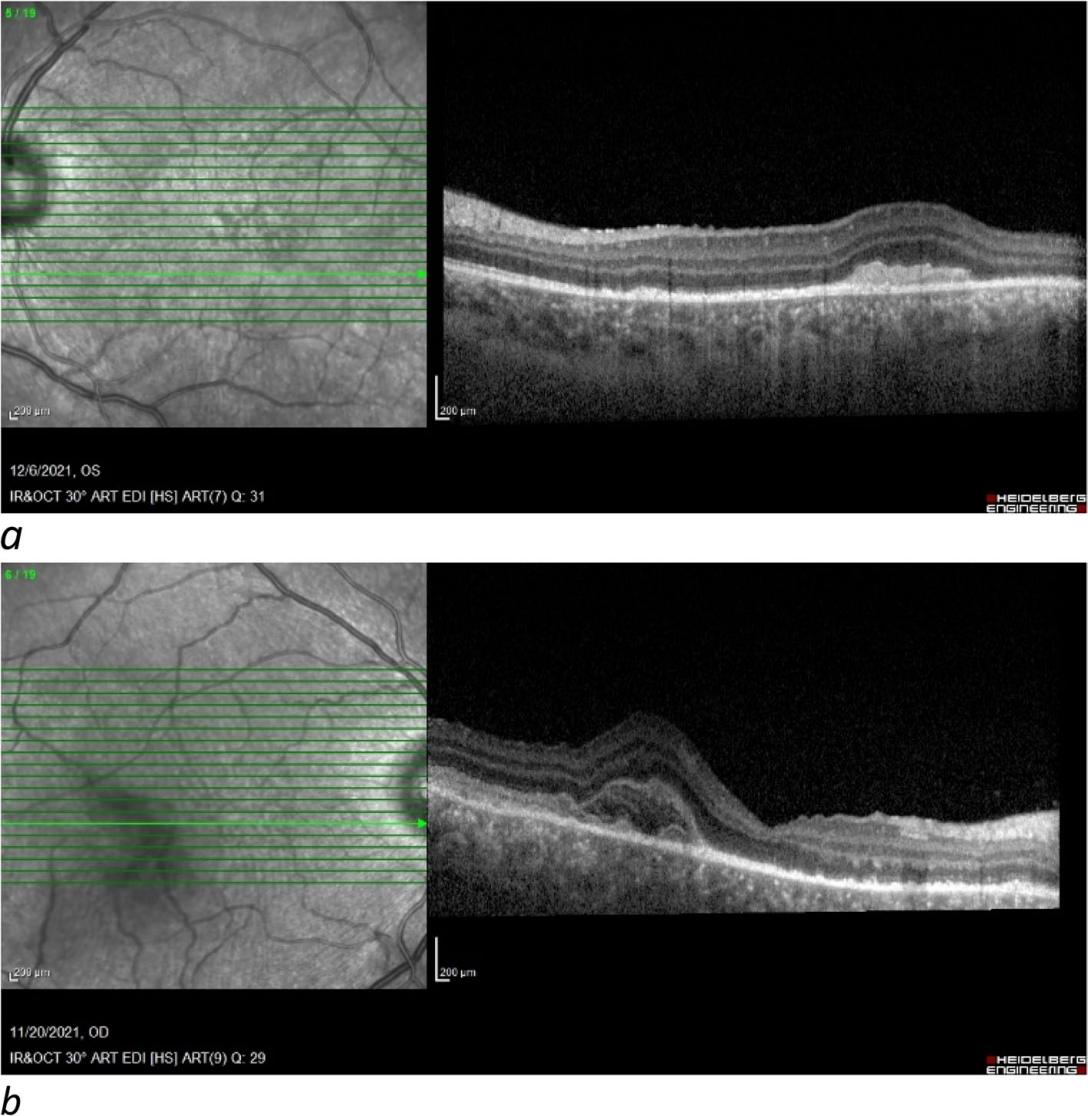
Macular OCT after treatment shows a decrease in subretinal fluid (**a**, **b**)

At the time of the last follow-up three weeks after treatment, BCVA was 20/20 and 20/30 in the right and left eye, respectively (Fig. 6).

## Discussion

Vaccine-related uveitis is a well-established and well-documented phenomenon and has been reported about all available vaccines. Although exact pathogenesis has remained unclear, molecular mimicry, antigen-specific cell, and antibody-mediated hypersensitivity reactions have been proposed as possible hypotheses [5].

Reports of post-COVID-19 immune-mediated diseases like uveitis, Guillain–Barre syndrome, or systemic lupus erythematosus have been published. It is postulated that COVID-19, the same as other viruses, can disturb self-tolerance and incite autoimmune responses through cross-reactivity with host cells. The same immunologic processes may occur following vaccination [6].

In the current article, we presented four cases of uveitis, including a case of VKH syndrome, after inoculation with inactivated COVID-19 Sinopharm vaccine. The first case was a patient with unilateral anterior uveitis and reactivated ulcerative colitis six days after receiving the second dose of the Sinopharm vaccine. The patient’s underlying disease had been inactive for four years without any medication. Ocular manifestations responded well to topical and systemic steroids, and systemic medications were initiated for treating active ulcerative colitis. In case 2, we observed the onset of bilateral anterior uveitis in an otherwise healthy female with no ocular history. Inflammation completely resolved after topical treatment and findings in our systemic workup were unremarkable. The third case developed panuveitis with two foci of posterior polar retinitis patches in the right eye and isolated anterior uveitis in the left eye. The patient responded to the combination of oral and topical steroids with complete resolution of uveitis and retinitis patches. Regarding the normal results of local and systemic evaluations in this patient, as well as the appropriate clinical response to the corticosteroid treatment, it can be concluded that the uveitis was a non-infectious immune-mediated type possibly triggered by the COVID vaccine.

The last case in this series was a young female diagnosed with probable VKH syndrome after inoculation with the second dose of inactivated vaccine. VKH is a multisystem immune-mediated disease presenting with bilateral, non-necrotizing granulomatous panuveitis, and serous retinal detachment associated with neurological, auditory, and/or dermatological disorders [7]. This condition is described as a T-cell mediated immune response triggered by environmental or immunogenetic agents; however, its exact etiology that primarily involves the choroid has not been recognized precisely. Genetic susceptibility and viral disease are probable risk factors of VKH [8].

There have been multiple reports of VKH syndrome following vaccination for influenza [9], yellow fever [10], and Bacillus Calmette–Guérin [11]. Papasavvas et al. reported reactivation of previously diagnosed and well-controlled VKH for the past six years and six weeks after inoculation with the second dose of the BNT162b2 vaccine [12]. Saraceno et al. described the onset of bilateral VKH in a 62-year-old healthy female following vaccination with Oxford-AstraZeneca chimpanzee adenovirus vectored vaccine [13]. To the best of our knowledge, this is the first reported case of VKH following vaccination with the inactivated vaccine of Sinopharm.

It has been suggested that dysregulation of the immune system and molecular mimicry are possible etiologies of uveitis following vaccination. Xin Le Ng et al. reviewed the ocular side effects of the COVID-19 vaccination. Regarding the overlap between the ocular manifestations of the vaccination and COVID-19 itself, researchers suggested a possible similar mechanism [14].

The appropriate response to corticosteroid treatment is the common denominator of all the introduced cases. The prompt response confirms that inflammation responds well to treatment without any significant vision-threatening complications, regardless of the type of uveitis. Therefore, ophthalmologists’ awareness of complications of the COVID-19 vaccine is important for reassuring patients regarding the benign course of inflammation and conducting specific research to identify the possible stimulating particle of the COVID-19 vaccine shortly. One possible mechanism of vaccine immunogenicity may be related to the adjuvant particles of vaccines that trigger immunologic reaction via multiple mechanisms, including antigen-presenting-cells activation, cytokine release, and sustained release of antigen due to depo effect of adjuvants, antibody production, and cellular recruitment [15].

Another responsible mechanism might be related to the existence of specific viral particles in vaccines, including the COVID-19 vaccine, which causes an immunologic reaction in the body, involving uveal tissues as potent immunogenic tissue in the eye.

It should be noted that no definite causal relationship can be established based on the cases in this report, and the possible ocular side effects are rare despite the large scale of worldwide vaccination. However, our cases and similar reports around the world raise concerns about the possibility of uveal inflammations in cases with a history of auto-immune systemic diseases or inactive uveitis after the COVID-19 vaccination.

## Data Availability

The datasets used during the current study are available from the corresponding author on reasonable request.
